# Comparison of three device generations of the StepWatch Activity Monitor: analysis of model version agreement in pediatric and adult independent ambulators

**DOI:** 10.3389/fspor.2024.1418018

**Published:** 2024-07-05

**Authors:** Wilshaw R. Stevens, Cody Barrett, Kelly A. Jeans

**Affiliations:** Movement Science Lab, Scottish Rite for Children, Dallas, TX, United States

**Keywords:** accelerometer, ambulatory activity, adults, pediatrics, reliability, agreement

## Abstract

**Purpose:**

Devices such as the StepWatch Activity Monitor (SAM) have been available for 20 years and have been shown to accurately measure ambulatory activity. This study aimed to evaluate the agreement among the three generations of the StepWatch Activity Monitor (SW3, SW4, and SW5) with respect to stride count.

**Methods:**

A total of 36 participants (age range, 6–55 years) participated in this institutional review board-approved study. The participants concurrently wore three different SAM model devices on the same leg and performed a 6-min walk test (6MWT). A research staff member of the laboratory manually counted the number of strides for the first 2 min of the test (2MWT). Agreement among the device models was evaluated by calculating ANOVAs and interclass correlation coefficients (ICCs) and creating Bland–Altman plots.

**Results:**

There was no significant difference among the model versions during the 6MWT and 2MWT (*p* > 0.05). The ICC for the total stride count was 0.993 (95% CI = 0.988–0.996) during the 2MWT and 0.992 (95% CI = 0.986–0.996) during the 6MWT. There was a near-perfect agreement (ICC ≥ 0.990) of each model version to the manually counted strides during the 2MWT. The systematic bias of all three SAM model versions was <1 step.

**Conclusions:**

The results from the present study demonstrate that the stride counts among all three devices are comparable and relative to the manual stride count. All three SAM model versions had an ICC of >0.90. Researchers can safely incorporate historical data from previous SAM model versions with newer data collected with the latest SAM model version.

## Introduction

Current research findings on the magnitude of physical inactivity in people of all ages are of great concern, and public health officials have developed multifaceted strategies to address this growing problem (1–5). In the last two decades, significant advancements have been made in research- and consumer-grade wearable sensor technology, allowing researchers to objectively measure physical activity daily and providing not only the granularity of data beyond the total volume of activity but also in-depth assessments of activity bout intensity and duration (6–10). Research-grade devices such as the StepWatch Activity Monitor (SAM; Modus Health, Edmonds, WA, USA) have been shown to accurately measure ambulatory activity. Research-grade devices walking speeds across different age groups and in individuals with various disabilities or diseases (8, 11–14).

The StepWatch Activity Monitor version 3 (SW3) was released in 2004 and has been used as a gold standard against various other devices/methods for objectively measuring daily ambulatory activity. Following its release in 2004, two additional versions of the device have been released, namely, version 4 (SW4) released in January 2018 and version 5 (SW5) released in October 2023. SW4 differs from SW3 in the way that it is initialized, as it allows the user to set up the device via Bluetooth and a mobile app. In addition, advancements were made in SW4 regarding the device's memory storage capacity and its ability to export ambulatory data per second, which was limited to every 3 s on SW3. The most recent device brought to market, SW5, has undergone significant technological advancements it is half the weight of its predecessor and can store data for up to a year (depending on the activity level of the user). In addition, the SW5 proprietary stride detection algorithm differs from all those of the previous models as it no longer utilizes a calibrated spring tensioner as part of its stride detection sensing capabilities.

To the best of our knowledge, no published research studies have evaluated the agreement among the three generations of the SAM. With the release of the latest model version (SW5), there is a possibility that all three versions of the device could be used in the field simultaneously. Not to mention, there is a need to investigate whether ambulatory activity data collected using the older version of the SAM is comparable to the data collected using the newest model version. Therefore, this study aimed to evaluate the agreement among three generations of the StepWatch Activity Monitor (SW3, SW4, and SW5) with respect to stride count in children and adults.

## Methods

This institutional review board-approved research study included a convenience sample of healthy pediatric and adult participants who were all independent ambulators. Informed consent was obtained from each participant, and assent was obtained where appropriate. Participant data were enrolled into a laboratory data registry, which was queried in order to perform the study analysis. The exclusion criteria included participants who were currently under treatment for an orthopedic injury, clinically diagnosed with a neurologic condition, non-ambulatory, unable to wear the activity monitor, and/or not able to follow directions.

The participants were recruited to perform a 6-min walk test (6MWT) and were fitted with three SAM devices. All three SAM devices were placed on the same leg (on either the right or left leg) in the following orientation: SW3, lateral ankle; SW4, medial ankle; and SW5, directly above the SW3 device (Supplementary Figure S1). Per the manufacturer documentation, SW3 or SW4 can be worn on the medial ankle for testing purposes. All SAM devices were initialized by entering the participant's height and selecting the default manufacturer settings.

After confirming that the participants were comfortable wearing the devices, they were asked to perform a 6MWT (15). The 6MWT course utilized in the present study was a rectangular shape rather than a linear 30-m course with marked end lines (15). The participants were instructed to walk as far as possible for 6 min, at their own comfortable pace (15). A single observer (a research staff member of the laboratory) recorded the time of day in seconds, in which the 6MWT started and ended. Previous studies have utilized a 2-min walk test (2MWT) to manually measure the accuracy of ambulatory activity data (13, 16). Therefore, the same single observer used a handheld tally counter to manually count the number of strides taken during the first 2 min of the test. Upon completion of the 6MWT, stride data were exported to a Microsoft Excel spreadsheet. For the SW3 devices, the StepWatch 3.1b software along with the SAM infrared docking station was utilized. The SW4 and SW5 devices were downloaded using the Bluetooth interface and the SAM mobile app. An epoch setting of a 10 s interval was selected for all SAM model versions as the SW3 device does not allow for second-by-second stride data exporting (17).

The data were analyzed for each participant, and inter-device model agreement was calculated on the total stride count. An ANOVA was run comparing the total stride counts among the three model versions during the 2MWT and 6MWT. For the 6MWT/2MWT data, agreement among the three device versions was evaluated by calculating intraclass correlation coefficients (ICC; two-way mixed model with the assumption of absolute agreement). Additionally, Bland–Altman plots with the 95% limits of agreement were created to assess the bias (mean differences) between the stride counts from each SAM model version and the manual stride count during the 2MWT portion of data (18, 19). All statistical analyses were run using SPSS (version 24, IBM Inc., Chicago, IL, USA) with statistical significance set at *α* = 0.05.

## Results

**Figure 1:**
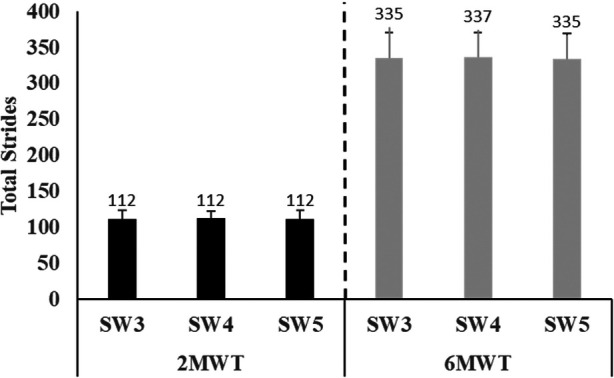
Total strides measured on the three model versions of the StepWatch Activity Monitor (SW3, SW4, and SW5) during the 2-min walk test (2MWT) and 6-min walk test (6MWT). No significant difference was observed among the devices during either test (*p* > 0.05).

A total of 36 participants (17 males/19 females; average age, 17 ± 11 years; range, 6–55 years) were included in this study (Supplementary Table S1). All the participants tolerated the testing conditions and were able to walk continuously for 6 min without taking breaks. The means and standard deviations for the total stride counts of the three SAM model versions (SW3, SW4, and SW5) during the 2MWT and 6MWT are presented in Figure 1. There was no significant difference in the total stride counts among all model versions for either testing condition (2MWT, *p* = 0.965; 6MWT, *p* = 0.965). The change in the number of strides detected between the different versions was assessed. When comparing SW3 and SW4, the individual difference between these two model versions ranged from −19 to 4 strides. When comparing SW3 and SW5, the individual difference between these two model versions ranged from −6 to 5 strides. Finally, when comparing SW4 and SW5, the individual difference ranged from −4 to 24 strides.

**Figure 2:**
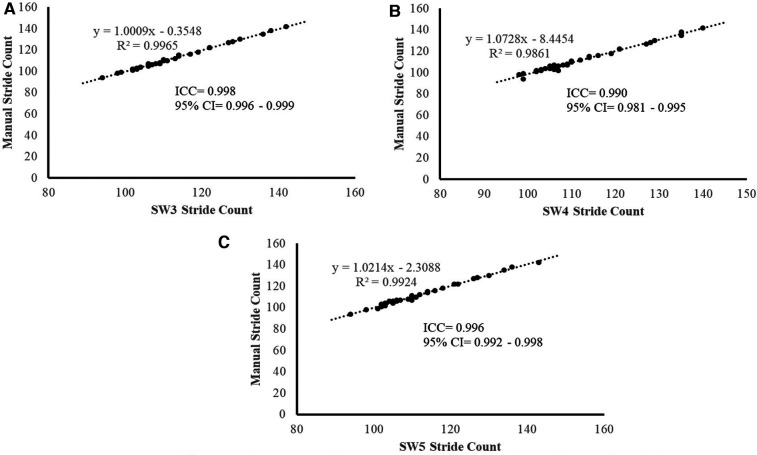
(**A–C**). Manual stride count vs. the StepWatch Activity Monitor stride count for the three versions [SW3, (**A**), SW4 (**B**), and SW5 (**C**)] used during 2-min walk test (2MWT). Near-perfect agreement between the manual counts and the devices measured stride counts (ICC > 0.990).

The ICC for the total stride count was 0.993 (95% CI = 0.988–0.996) during the 2MWT and 0.992 (95% CI = 0.986–0.996) during the 6MWT (Table 1). Figures 2A–C illustrate the near-perfect agreement (ICC ≥ 0.990) of each model version to manually counted strides during the 2MWT.

**Table 1:** Interclass correlation coefficient (ICC) with 95% CI of the total stride counts among the three model versions of the StepWatch Activity Monitor: SW3, SW4, and SW5. There was near-perfect agreement among the three model versions of the StepWatch Activity Monitor during the 2-min walk test (2MWT) and 6-min walk test (6MWT).

	ICC	95% CI
2MWT—SW3, SW4, SW5	0.993	0.988–0.996
6MWT—SW3, SW4, SW5	0.992	0.986–0.996

**Figure 3:**
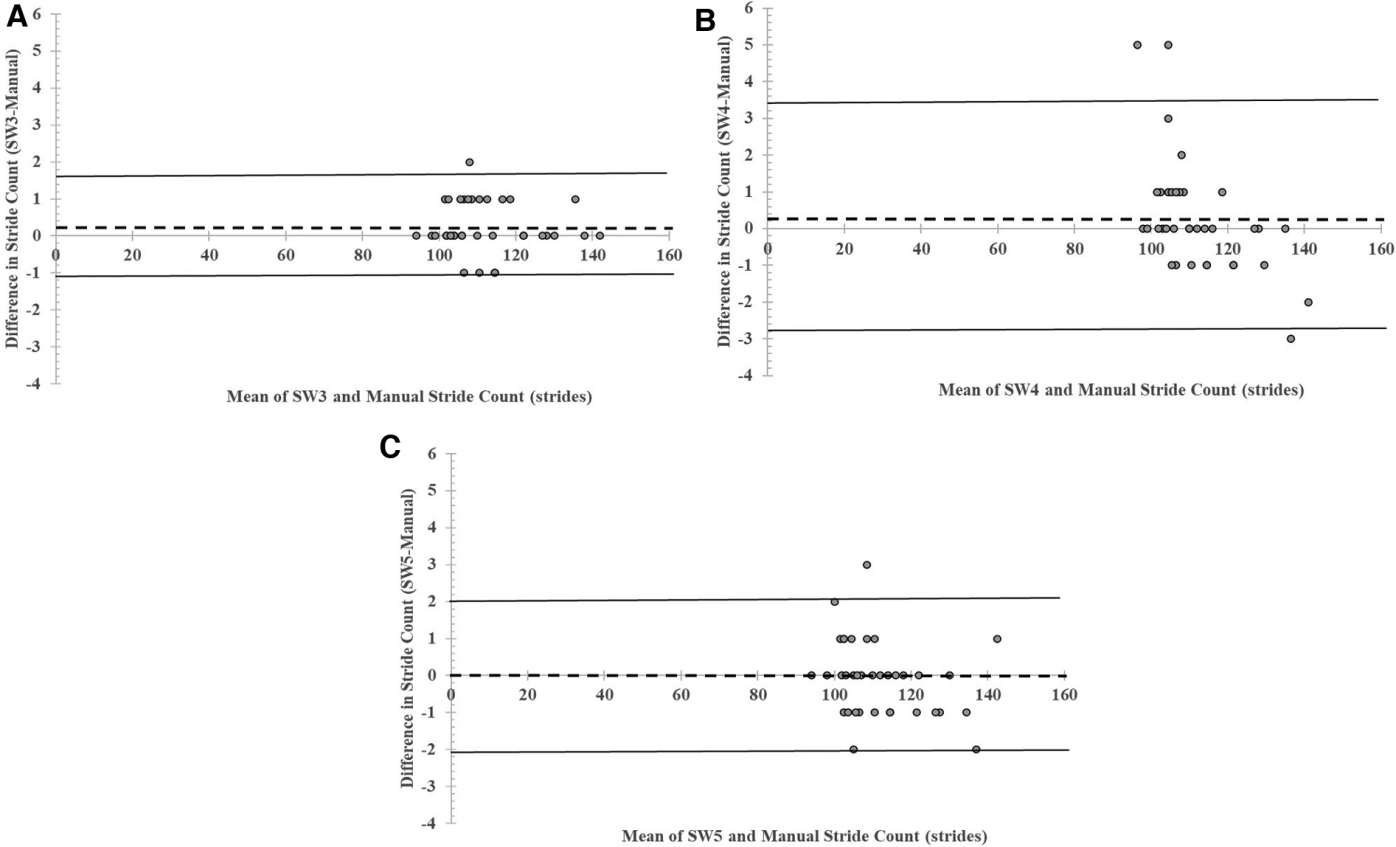
(**A–C**). Mean bias and 95% limits of agreement for stride count recorded by the three StepWatch Activity Monitor model version [SW3 (**A**), SW4 (**B**), and SW5 (**C**)] compared to manual stride count measured during a 2-min walk test (2MWT).

Figures 3A–C display the Bland–Altman plots depicting the mean bias and 95% limits of agreement for the total stride counts of the three model versions of the SAM relative to the manual stride count during the 2MWT. The systematic bias of all three SAM model versions was <1 step and near zero for SW5 (Figure 3C). In addition, 95% limits of agreement for the three SAM model versions were as follows: −1.1 to 1.6 strides (SW3), −2.9 to 3.4 strides (SW4), and −2.1 to 2.0 (SW5). Bland–Altman plots depicting the percentage difference bias and 95% limits of agreement are displayed in Supplementary Figures S2A–C. The systematic bias of the percentage difference of all three SAM model versions was <1% (Supplementary Figures S2A–C).

## Discussion

The present study directly compared the agreement of three model versions of the SAM in children and adults. As the SAM continues to be used by researchers/clinicians, it is important to understand how reliable these model versions are in producing similar results, interchangeably. The results from the present study demonstrate that stride counts among all three devices are highly comparable. Utilizing the agreement ratings proposed by Landis and Koch (20), there was a near-perfect agreement between the three SAM model versions for both the 2MWT and 6MWT . The ICCs for stride count during the 2MWT and 6MWT were 0.993 and 0.992, respectively. In addition, relative to the manual stride count, all three SAM model versions had an ICC of >0.90.

There is precedence for comparing wearable sensor model versions, as researchers have assessed differences in commonly used research-grade physical activity monitors such as the ActiGraph (ActiGraph, Pensacola, FL, USA) and the activPAL (PAL Technologies Ltd., Glasgow, UK) (21, 22). To the best of our knowledge, prior to the current study, this analysis has not been done across multiple SAM model versions. Previous studies have primarily been conducted with the older version of the StepWatch Activity Monitor. A study by McDonald et al. (23), conducted on subjects of varying ages and walking speeds during a 10-min walking test, demonstrated an accuracy rate of 99.87% compared to observer-counted steps. Similar study designs performed in individuals with a unilateral transtibial amputation and a large cohort of inpatients admitted to a rehabilitation unit showed that during a 6MWT, the SAM compared to a manual stride count had an ICC of >0.90 (24, 25). In the present study during the 2MWT, similar findings were found when assessing all three SAM model versions (Figures 2A–C). The Bland–Altman plots revealed an average difference of <1 stride compared to the manual stride count and limits of agreement not exceeding three strides for either SAM model version (Figures 3A–C). These findings are similar to previous literature that reported an average difference of 1.6 strides and a 95% CI of −0.29 to 3.44 strides in the SW3 model version (13).

A novel contribution of this study is not only the comparison across three SAM model versions but also the incorporation of SW5, which is the newest model version commercially available. Based on the results of this study, SW5 provides sufficient accuracy and compares favorably to SW3, which remains the most prominently used SAM model version. In addition, there was a lack of evidence prior to this study on the comparability of SW4 to SW3, which was the intermediate device released in 2018. When assessing the individual differences in the number of strides taken, it is interesting to note that comparisons to SW4 had the highest range of differences compared to the SW3 and SW5 versions (although no statistically significant difference was observed among all three devices; Figure 1). As stated previously, SW4 differs from SW3 in the way that is initialized, as its internal component includes the hardware necessary for Bluetooth connections along with the advancements in its memory storage capacity. However, this does not fully explain why this model version in our testing environment appeared to be different in some individual cases. In our study design, SW4 was always worn on the medial side of the participant's leg, and although this is appropriate for conducting the testing of the SAM devices per the manufacturer, there is the possibility that this arrangement may have contributed to the wide range of differences observed. Our study did not randomize the location of these devices on the participant's leg, and future studies should consider doing so.

The present study had limitations that should be considered. The oldest version of the SAM utilized in the present study was SW3, which was released to the market in 2004. However, the timing is not entirely clear on whether there were devices commercially available between when the original SAM patent was filed in 1996 and the article by Coleman et al. in 1999 outlining the device and its capabilities (11, 26). A basic search for published articles between 1996 and 2003 mentioned that the SAM does yield some results and therefore additional analysis would need to be done to investigate the comparability of those devices to SW3.

Our study assessed agreement among the SAM model versions in a controlled laboratory environment during a specific walking protocol using a single observer to manually count strides. The present study included children and adult participants across a wide age range, and therefore average walking speed during the 6MWT spanned a wide range of walking speeds (range, 0.96–1.51 m/s). A significant advantage of the SAM over commercial-grade activity monitors has been its ability to accurately detect ambulatory activity across a wide range of speeds (27). However, our study does not answer the question of whether these findings translate to a free-living environment. Further investigations should consider assessing a free-living environment, which would include a host of additional factors such as intermittent walking bouts, rest periods, and a wide range of walking speeds. In addition, a more rigorous laboratory testing protocol would also have more than one independent observer to manually count the number of strides taken and a video recording for verification purposes.

Additional study design concerns will need to be addressed in subsequent studies as continuous wear of the SAM on the medial side of the leg may introduce discomfort to some participants. In the present study, the first 2 min of the 6MWT was considered a 2MWT and utilized to perform a manual count of the number of strides taken. It is reasonable to expect that if the participants are told that they were ambulating for only 2 min, their walking pace may increase and therefore the total number of strides reported in the present study may be different. Finally, SW5 is marketed by the manufacturer as a major technological advancement of the SAM, as it includes a new proprietary stride detection algorithm. As this is a proprietary algorithm, it is up to the research community to test this device in a wide range of use cases, to get a better understanding of its strengths and weaknesses.

In conclusion, stride counts measured by the StepWatch Activity Monitor versions 3, 4, and 5 demonstrate strong agreement. Data collected from these three model versions can be combined in their analysis. As the latest version of the SAM (SW5) gains popularity, researchers can safely incorporate historical data from previous SAM model versions with newer data collected with the latest SAM model version.

## Data Availability

The raw data supporting the conclusions of this article will be made available by the authors, without undue reservation.
