# Supplementary material for: Comparison of three device generations of the StepWatch Activity Monitor: analysis of model version agreement in pediatric and adult independent ambulators
Source: Front Sports Act Living. 2024 Jul 5;6:1418018. doi: 10.3389/fspor.2024.1418018 (PMC11257887; doi:10.3389/fspor.2024.1418018)
Supplement: Supplementary file 1 [file Table1.docx]

Supplementary Table 1. Participant demographics (N=36; age range 6- 55 years); Six minute walk test (6MWT)

|  | N |
| --- | --- |
| Height (meters) | 1.60±0.17 |
| Sex (Males/Females) | 17/19 |
| Age (years) | 17±11 |
| 6-12 | 15* |
| 13-19 | 11* |
| 20+ | 10* |
| 6MWT (meters) | 450.7±66.3 |

* Indicates participant count in each age group.
