# Supplementary figures and images for: Comparison of three device generations of the StepWatch Activity Monitor: analysis of model version agreement in pediatric and adult independent ambulators
Source: Front Sports Act Living. 2024 Jul 5;6:1418018. doi: 10.3389/fspor.2024.1418018 (PMC11257887; doi:10.3389/fspor.2024.1418018)

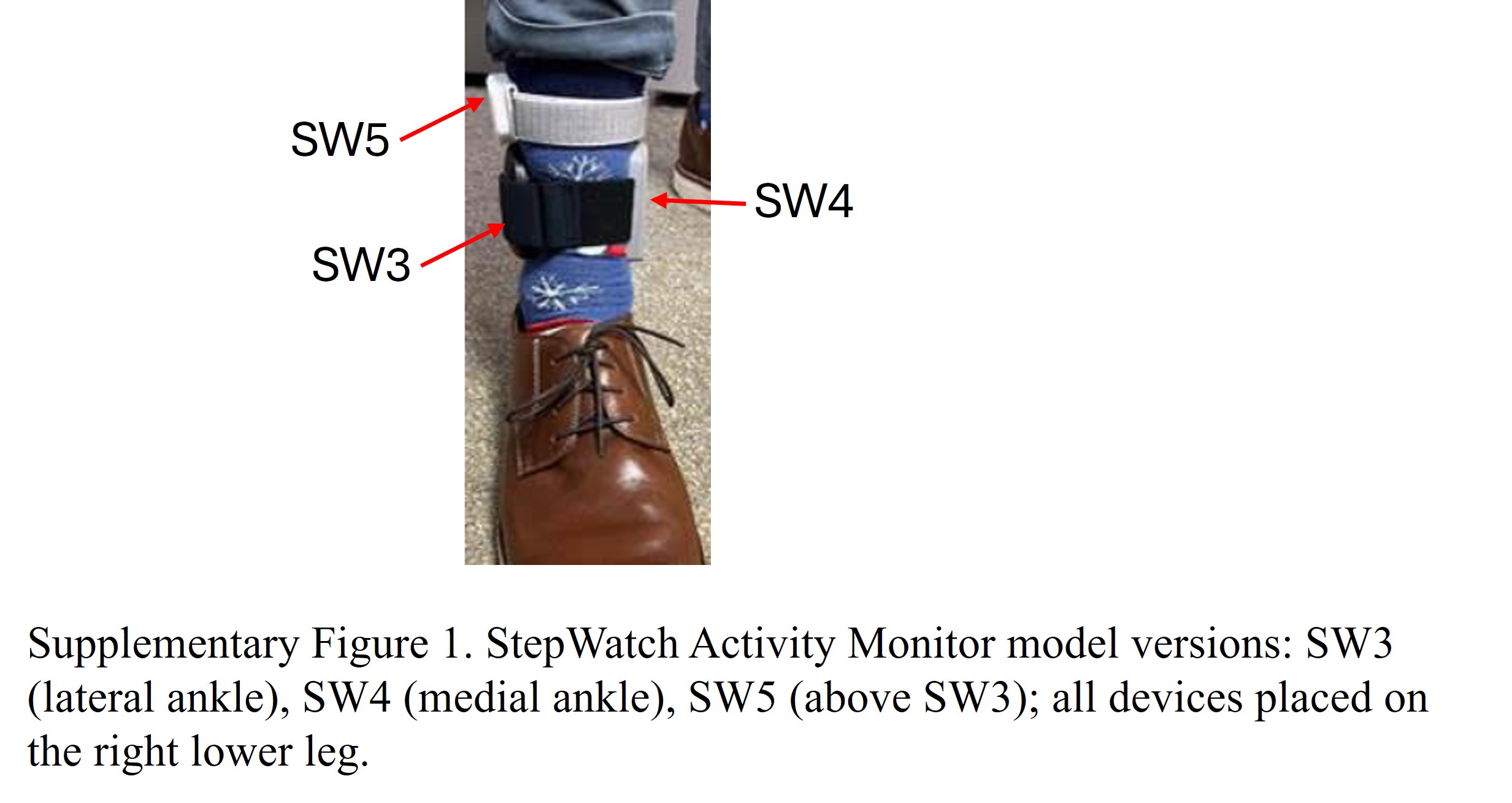

Supplement: Supplementary file 2 [file Image1.jpeg]

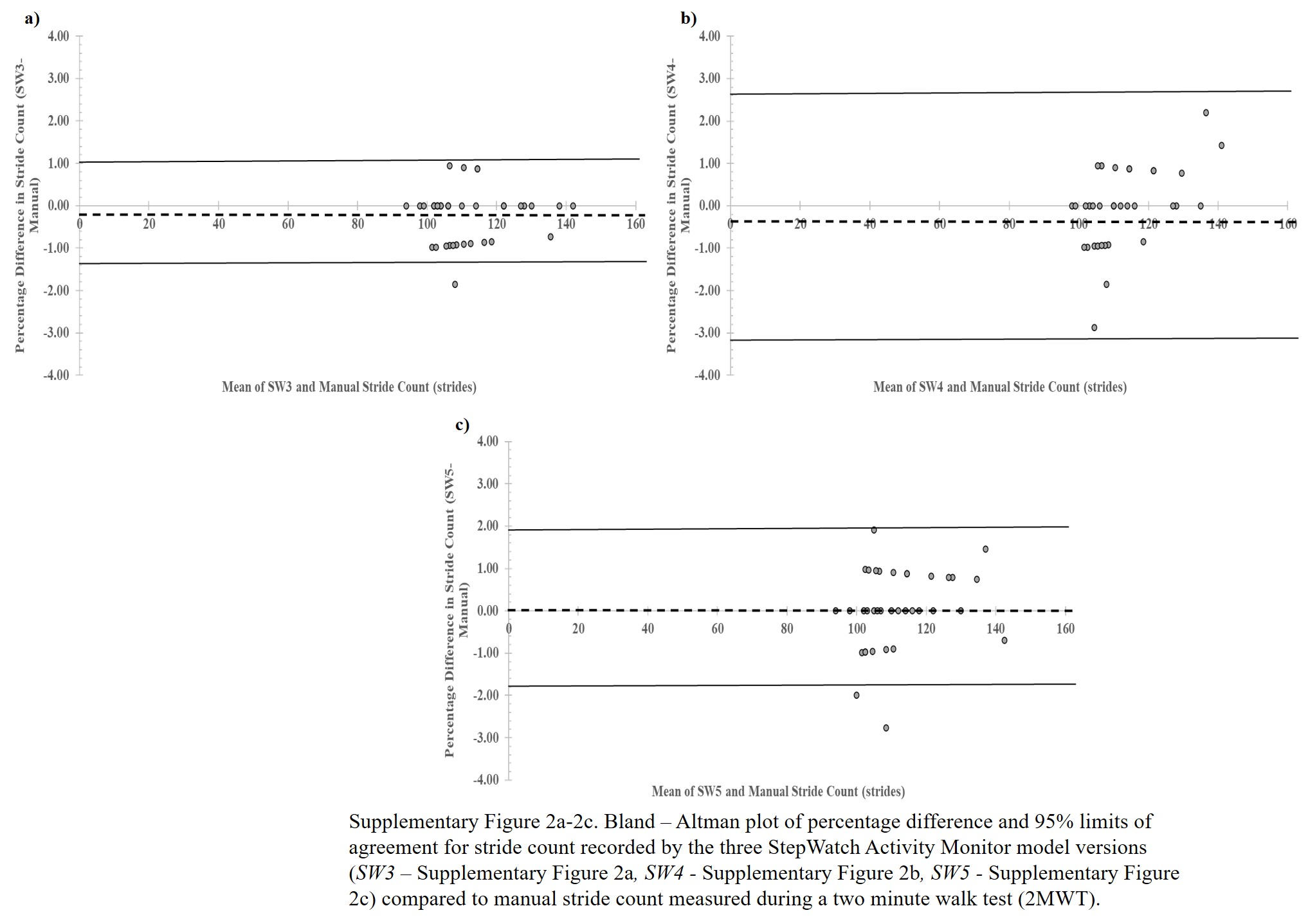

Supplement: Supplementary file 3 [file Image2.jpeg]
